# Erectile Dysfunction as a Manifestation of Newly Diagnosed Diabetes: A Case Report

**DOI:** 10.7759/cureus.95893

**Published:** 2025-11-01

**Authors:** Marta Moreira Reis, Carla Marques, Bárbara Morais Costa, Francisca Fontes, Sandrina Rodrigues, Ana Isabel Silva

**Affiliations:** 1 Family Medicine, ULS Médio Ave, USF São Tomé, Santo Tirso, PRT

**Keywords:** case report, diabetes complications, diabetes mellitus, erectile dysfunction, microangiopathy, microvascular complications

## Abstract

The prevalence of diabetes mellitus (DM) continues to rise worldwide. One possible complication of this condition is microangiopathy, with erectile dysfunction (ED) being a common example. The impact of DM on ED is significant, as it is estimated that more than half of men with type 2 diabetes (T2D) suffer from this condition. Furthermore, the likelihood of a man with DM developing ED is much higher than that of a non-diabetic man. We present the case of a 39-year-old man who sought medical consultation for ED that had been progressing for two months. His medical history was unremarkable, and he was not taking any regular medication. He is the father of six children. Using the International Index of Erectile Function - 5 (IIEF-5) to assess ED, he was classified as having “severe erectile dysfunction”. Physical examination was unremarkable except for being overweight (body mass index: 27.1 kg/m²). Laboratory tests revealed slightly low total and free testosterone levels and a fasting glucose level of 146 mg/dL. Repeat testing confirmed elevated glucose levels, with a hemoglobin A1c of 6.9%. A diagnosis of DM was established, and the patient started metformin, which led to improvement of his initial complaint. This case highlights that when treating a patient with ED, it is essential to perform a thorough differential diagnosis that includes major causes of this condition, particularly DM. The earlier DM treatment is initiated, the sooner complications such as ED can be prevented. Family physicians play a crucial role in identifying and addressing this issue, not only because it is often underreported but also because it may serve as an early marker of cardiovascular risk.

## Introduction

The prevalence of diabetes mellitus (DM) has been increasing both globally and nationally. In 2016, the World Health Organization (WHO) reported in its Global Report on Diabetes that the prevalence of DM had nearly doubled between 1980 and 2014, rising from 4.7% to 8.5% [1]. Similarly, Portugal’s Direção-Geral da Saúde (DGS) stated in the 2023 Annual Report of the National Diabetes Observatory that the national prevalence of DM increased from 13.7% to 14.1% between 2019 and 2021 [2].

According to the American Urological Association, erectile dysfunction (ED) is defined as the inability to achieve or maintain an erection sufficient for satisfactory sexual performance [3]. Although ED can have multiple contributing factors, including hormonal and vascular mechanisms, the interplay between insulin resistance and testosterone levels is particularly relevant in diabetic patients. One of the well-known complications of DM is microangiopathy [4,5]. In patients with insulin resistance, there is increased degradation and decreased synthesis of nitric oxide (NO), which is essential for vasodilation. Consequently, one of the outcomes of this mechanism may be ED, as the absence of NO leads to vasoconstriction of the arteries, arterioles, and sinusoids of the corpus cavernosum [4,5]. Moreover, there is a known association between hypogonadism and type 2 diabetes (T2D), since low testosterone levels may contribute to insulin resistance [4]. Studies have shown that approximately 40% of men with T2D have low testosterone levels, and nearly all of them experience ED [4].

The impact of DM on ED is highly significant, as it is estimated that more than half of men with type 2 DM suffer from this condition [4,6]. Furthermore, the likelihood of developing ED is considerably higher in men with DM compared to non-diabetic men [6].

## Case presentation

On November 24, 2023, a 39-year-old man presented to a scheduled consultation with his wife, reporting new-onset ED with a two-month history. He had no regular medical follow-up prior to this episode. The patient is married, has six children, and works as a furniture assembler. At the time of the consultation, he had become a father three months earlier.

The patient had no prior medical history as he took no regular medication, had not been diagnosed with any chronic condition, and had no surgical history. He was a smoker with a 30 pack-year history, denied alcohol or drug use, and reported no regular physical activity. His diet was varied, including soup and fruit a few times per week. As for family history, he reported that his father had type 2 DM, with no other relevant familial conditions, including no history of cancer.

He denied urinary symptoms such as changes in urinary flow or dysuria. He also denied visual changes, limb pain, paresthesia, or claudication, as well as symptoms suggestive of cardiovascular disease such as chest pain, palpitations, or dyspnea. Likewise, he denied endocrine-related symptoms such as weight or appetite changes, alterations in hair distribution, or changes in temperature sensitivity.

The patient reported a consistent inability to achieve erections, including the absence of morning erections. He did not identify any triggering factors, nor factors that alleviated or worsened the condition. He denied changes in libido and had never experienced similar symptoms in the past. As contraception, the couple used a progestin-only pill and condoms while his wife awaited tubal ligation.

The International Index of Erectile Function - 5 (IIEF-5) questionnaire was used to assess the degree of ED, yielding a score of six, consistent with severe ED.

On physical examination, his body mass index was 27.1 kg/m², blood pressure 127/81 mmHg, and heart rate 100 bpm. The neurological examination was unremarkable, and the genital exam revealed no penile deformities or other relevant findings.

Laboratory tests were ordered, including fasting glucose, lipid profile, total and free testosterone, thyroid function, and prolactin. Referral to urology was considered pending the results, and a psychology consultation was suggested, which the patient postponed.

On December 20, 2023, he returned with results from November 29, 2023, which revealed: TSH and prolactin within normal ranges; slightly decreased total testosterone (2.68 ng/mL) and free testosterone (0.64 ng/mL); fasting glucose: 146 mg/dL.

The patient confirmed fasting prior to blood collection.

As the diagnostic criteria for DM require confirmation of hyperglycemia on a separate occasion, repeat testing was ordered, including fasting glucose and glycated hemoglobin (HbA1c). On January 10, 2024, results showed: fasting glucose 166 mg/dL and HbA1c 6.9%. A diagnosis of DM was therefore established. Metformin 1000 mg once daily was initiated, along with dietary and physical activity counseling.

He was referred to urology and endocrinology. At present, he remains under endocrinology follow-up. His glycemic control has improved, with an HbA1c <6,5% and stable testosterone levels. He did not attend the urology appointment due to the resolution of his primary symptom - ED.

## Discussion

The confirmation of an ED diagnosis should take into account its main potential causes [7]. In clinical practice, it is essential to distinguish between organic and psychogenic causes of ED [7]. Hormonal disorders, vascular disease, neurological conditions, medication side effects, and psychological stress may all contribute to varying degrees [7]. In this case, the absence of psychological or neurological symptoms and the presence of hyperglycemia suggested a predominantly metabolic and vascular etiology.

DM induces a state of endothelial dysfunction driven by chronic hyperglycemia, oxidative stress, and inflammation [4,5]. This state impairs NO bioavailability and increases the expression of vasoconstrictors such as endothelin-1 [4,5]. This imbalance between vasodilators and vasoconstrictors, together with a pro-thrombotic and pro-inflammatory state, leads to progressive microvascular damage and impaired smooth muscle relaxation within the corpus cavernosum [4,5]. Beyond ED, DM is associated with several well-known microvascular and macrovascular complications that share common pathophysiological mechanisms, including retinopathy, nephropathy, neuropathy, coronary artery disease, and peripheral arterial disease [4,5]. Patients with newly diagnosed diabetes should promptly undergo monitoring for these conditions. Recognizing ED as part of this spectrum reinforces the importance of early glycemic control and regular follow-up for other diabetic complications [4,5].

To assess ED, validated questionnaires such as the International Index of Erectile Function-5 (IIEF-5) questionnaire may be used [7,8]. This tool evaluates five domains of sexual function, including confidence in achieving and maintaining an erection, frequency and rigidity of erections during sexual stimulation, ability to sustain an erection after penetration, difficulty in maintaining an erection until completion of intercourse, and overall satisfaction with sexual activity. Each question is scored from 1 to 5, with a total possible score ranging from 5 to 25. Scores between 22 and 25 indicate no ED, 17-21 mild to moderate, 8-11 moderate, and 5-7 severe dysfunction [7,8]. In this case, the patient scored 6, consistent with severe ED.

Treatment of DM-induced ED is, like its pathophysiology, complex and therefore requires a holistic approach [4,5]. In simple terms, glycemic control may help treat and/or prevent DM complications such as ED [5]. Studies have shown that oral antidiabetic agents, particularly metformin, have a positive impact on the management of ED [4]. The beneficial effects of metformin are related both to the reduction of insulin resistance and the improvement of vasodilation, with an additional protective role against endothelial dysfunction [4]. Consequently, it may also have a positive effect on hypogonadism that can be associated with DM [4].

Phosphodiesterase-5 (PDE-5) inhibitors are also a valid and safe therapeutic option in these cases [4,5]. Unfortunately, they tend to be less effective in diabetic men than in non-diabetic men because reduced NO availability and structural vascular changes often limit the efficacy of these pharmacological therapies [4,5].

Other therapeutic alternatives include intracavernosal injections, vacuum constriction devices, and penile injections [4,5].

As expected, non-pharmacological interventions - such as lifestyle modifications (smoking cessation, dietary changes, and regular physical activity) - are also essential [4,9]. Smoking contributes to endothelial dysfunction, which can further exacerbate ED in diabetic patients. Addressing smoking habits is therefore a key component of management and prevention [4,5].

## Conclusions

As the prevalence of diabetes continues to rise worldwide, its complications are also becoming increasingly common. ED is a frequent yet often underrecognized complication of DM that can cause significant distress for the patient. Early recognition of ED as a possible initial manifestation of DM may allow timely diagnosis and initiation of appropriate metabolic control, thereby preventing further microvascular and macrovascular complications.

Although ED typically develops later during the course of diabetes, this case illustrates that microvascular complications may occasionally present at a younger age, even before metabolic decompensation is evident and with minimal warning signs.

Therefore, it is crucial that family physicians actively inquire about this condition. Addressing ED not only improves patients’ psychosocial well-being but also provides an opportunity for cardiovascular risk assessment and holistic management of chronic disease.

## References

[1] (2025). World Health Organization: Global Report on Diabetes. World Health Organization.

[2] (2025). Direção-Geral da Saúde. National Program for Diabetes - Challenges and Strategies. Available from. https://www.dgs.pt.

[3] Burnett AL, Nehra A, Breau RH (2018). Erectile Dysfunction: AUA Guideline. J Urol.

[4] Christoforidis A, Georeli I, Dimitriadou M, Galli-Tsinopoulou A, Stabouli S (2022). Arterial stiffness indices in children and adolescents with type 1 diabetes mellitus: A meta-analysis. Diabetes Metab Res Rev.

[5] Kamenov ZA (2015). A comprehensive review of erectile dysfunction in men with diabetes. Exp Clin Endocrinol Diabetes.

[6] Kouidrat Y, Pizzol D, Cosco T (2017). High prevalence of erectile dysfunction in diabetes: A systematic review and meta-analysis of 145 studies. Diabet Med.

[7] (2025). UpToDate: Evaluation of male sexual dysfunction. https://www.uptodate.com/contents/evaluation-of-male-sexual-dysfunction.

[8] Pechorro PS, Calvinho AM, Pereira NM, Vieira RX (2011). Validation of a Portuguese version of the International Index of Erectile Function-5 (IIEF-5). (Article in Portuguese). Rev Int Androl.

[9] Hadisuyatmana S, Malik G, Efendi F, Reisenhofer S, Boyd J (2023). The experiences and barriers in addressing type 2 diabetes mellitus-associated erectile dysfunction: A mixed-method systematic review. Syst Rev.

